# Exploring ChatGPT’s Efficacy in Orthopaedic Arthroplasty Questions Compared to Adult Reconstruction Surgeons

**DOI:** 10.1016/j.artd.2025.101772

**Published:** 2025-07-14

**Authors:** Benjamin Nieves-Lopez, Clayton Wing, Bryan D. Springer, Keith T. Aziz

**Affiliations:** a University of Puerto Rico, Medical Sciences Campus, San Juan, Puerto Rico; b Department of Orthopedic Surgery, Mayo Clinic Florida, Jacksonville, FL

**Keywords:** ChatGPT 4, Arthroplasty, Adult reconstruction, Orthopaedic education

## Abstract

**Background:**

Chat Generative Pre-trained Transformer (ChatGPT) is a language model designed to conduct conversations utilizing extensive data from the internet. Despite its potential, the utility of ChatGPT in orthopaedic surgery, particularly in arthroplasty, is still being investigated. This study assesses ChatGPT’s performance on arthroplasty-related questions in comparison to an Adult Reconstruction Fellow and a Senior level attending.

**Methods:**

A total of 299 questions from the Adult Reconstruction self-assessment on OrthoBullets were evaluated using ChatGPT 4. Performance was analyzed across different question categories and compared with the performance of an Adult Reconstruction Fellow and Senior level attending arthroplasty surgeon with a *Chi*-square test. Further comparisons were performed to assess ChatGPT’s accuracy rate on image-based questions. Statistical significance was set to a *P* value ≤ .05.

**Results:**

ChatGPT achieved a 66.9% accuracy rate compared to 84.3% and 85.3% obtained by the Fellow and Attending, respectively. No significant differences in performance were observed across question categories. ChatGPT demonstrated better results in text-only compared to image-based questions. Although not statistically significant, ChatGPT showed the highest accuracy rate in questions that included both an X-ray and a clinical picture.

**Conclusions:**

ChatGPT performed inferior to an Adult Reconstruction Fellow and Attending and it provided more accurate answers when prompted with text-only questions. These findings suggest that while ChatGPT can serve as a useful supplementary resource for arthroplasty topics, it cannot substitute for the clinical judgment required in detailed assessments. Further research is necessary to optimize and validate the use of artificial intelligence in medical education and patient care.

## Introduction

Chat Generative Pre-trained Transformer (ChatGPT) is a large language model developed by OpenAI that mimics human-like dialog based on vast internet resources and refines its capabilities through iterative learning [1]. Since its release, ChatGPT has been rapidly integrated across various sectors, including health care, where its potential to assist in medical education and clinical decision-making is being keenly explored [[2], [3], [4]]. The adoption of artificial intelligence (AI) tools, such as ChatGPT, in medicine has demonstrated significant benefits not only in educational contexts but also for health-care professionals and patient interactions [[3], [4], [5]].

Notably, ChatGPT has achieved impressive results on standardized medical examinations such as the United States Medical Licensing Examination, achieving scores indicative of substantial medical knowledge and reasoning capability [6]. These achievements highlight the evolving utility of ChatGPT in medical training and patient care, prompting further research into its application across various medical disciplines [3,[5], [6], [7], [8]]. In the field of orthopaedics, the capabilities of ChatGPT 3.5 and 4 have been examined in relation to specialty and subspecialty board examinations. Studies have shown that ChatGPT 3.5 and ChatGPT 4 would most likely fail to pass the American Board of Orthopaedic Surgery examinations [5,9]. However, subsequent studies indicate that ChatGPT 4 demonstrates enhanced performance, suggesting a positive correlation between technological advancements and improved outcomes in orthopaedic examinations [[8], [9], [10], [11]].

The integration of ChatGPT in orthopaedic practice, particularly in arthroplasty, has demonstrated multiple advantages. It has proven effective in delivering accurate, evidence-based responses to frequently asked patient questions regarding procedures like total hip arthroplasty (THA), offering answers that patients feel more comfortable with compared to information provided by arthroplasty-trained nurses [4,12]. Additionally, the model's ability to create educational summaries pertinent to patient care in arthroplasty enhances its value as an educational tool for patients and health-care professionals [13]. Despite these advances, the extent to which ChatGPT can accurately respond to arthroplasty-specific inquiries, particularly those involving images, remains unknown.

This study aimed to evaluate ChatGPT's accuracy in answering arthroplasty-related questions, compare its performance with that of an Adult Reconstruction Fellow and Attending, and explore its performance in image-based questions.

## Material and methods

This study employed Adult Reconstruction of the Hip and Knee self-assessment questions from educational website OrthoBullets (www.orthobullets.com) to evaluate the performance of ChatGPT 4, a commercially available large language model. A comprehensive dataset of 299 questions, each accompanied by multiple-choice answers, was methodically entered into ChatGPT's text box. Accompanying visual elements such as images, tables, charts, and graphs were copied directly from the web page and pasted into ChatGPT's text box in the original JPG format to ensure no changes in resolution or pixelation. To eliminate any potential learning bias from previous interactions, prior conversations with ChatGPT were deleted, and a new chat session for each question was initiated. After presenting each question and its choices, ChatGPT automatically selected one of the provided choices as the answer. If ChatGPT failed to provide a singular, clear response or offered multiple responses, it was prompted to "Choose 1 from 1 to 5." This directive was needed for only 16 questions (5.3%), all of which were answered on the subsequent prompt.

To explore potential variations in ChatGPT's performance across different medical topics, the questions were classified into 10 specific categories: anatomy (9 questions, 3.0%), arthroplasty trauma (30 questions, 10.0%), basic science (42 questions, 14.0%), medical management (21 questions, 7.0%), miscellaneous (16 questions, 5.4%), periprosthetic joint infection (PJI) (23 questions, 7.7%), primary THA (43 questions, 14.4%), primary total knee arthroplasty (TKA) (56 questions, 18.7%), revision THA (38 questions, 12.7%), and revision TKA (21 questions, 7.0%). This classification system was devised and validated by 2 board-certified orthopaedic surgeons. Any discrepancies in categorization were addressed through collaborative review and discussion, ensuring an accurate assignment of categories.

Image-based questions (168 questions) were stratified into subcategories based on the type of visual elements they contained, allowing for a more detailed analysis of performance variations. These were divided into clinical picture (20 questions, 11.9%), histology (3 questions, 1.8%), magnetic resonance imaging (MRI) (3 questions, 1.8%), ultrasound (1 question, 0.6%), X-ray (123 questions, 73.2%), X-ray and clinical picture (11 questions, 6.5%), X-ray and computed tomography (CT) (2 questions, 1.2%), and X-ray and MRI (5 questions, 3.0%).

In addition to assessing ChatGPT, the same set of questions was administered to 2 board-certified orthopaedic surgeons, 1 current adult reconstruction fellow, and 1 fellowship-trained senior level adult reconstruction attending in practice for 20 years to serve as a comparative performance baseline.

### Data analyses

*Chi*-square tests were conducted to compare the overall performance between ChatGPT 4 and the adult reconstruction fellow and attending. The performances of ChatGPT 4, the adult reconstruction fellow, and the attending were independently analyzed and compared across different question categories using a *Chi*-square test to identify any variations in response accuracy based on the question topic. Additionally, separate analyses were employed to evaluate ChatGPT 4's accuracy on questions that included visual elements, as opposed to text-only questions. Further stratification of the visual elements was performed to assess ChatGPT 4's variations in performance. All statistical analyses were performed using SPSS software, version 29.0, with a threshold for statistical significance set at a *P* value ≤ .05.

This study did not involve human or animal subjects. The analysis utilized deidentified, standardized questions from OrthoBullets, ensuring that no personal or sensitive data were collected. Therefore, institutional review board approval was not required.

## Results

### Performances of ChatGPT, adult reconstruction fellow, and attending by question category

ChatGPT 4 accurately answered 77.8% of the Anatomy questions (7 of 9 questions), 60.0% (18 of 30 questions) in Arthroplasty Trauma, 83.3% (35 of 42 questions) in Basic Science, 66.7% (14 of 21 questions) in Medical Management, 75.0% (12 of 16 questions) in Miscellaneous, 73.9% (17 of 23 questions) in PJI, 58.1% (25 of 43 questions) in Primary THA, 67.9% (38 of 56 questions) in Primary TKA, 55.3% (21 of 38 questions) in Revision THA, and 61.9% (13 of 21 questions) in Revision TKA, as demonstrated in Table 1. No significant differences were observed in the performance of ChatGPT 4 across the different categories (*P* value = .255).

**Table 1:** ChatGPT 4 performance.

Question category	Correct (n = 200) n (%)	Incorrect (n = 99) n (%)
Anatomy	7 (77.8)	2 (22.2)
Arthroplasty trauma	18 (60.0)	12 (40.0)
Basic science	35 (83.3)	7 (16.7)
Medical management	14 (66.7)	7 (33.3)
Miscellaneous	12 (75.0)	4 (25.0)
PJI	17 (73.9)	6 (26.1)
Primary THA	25 (58.1)	18 (41.9)
Primary TKA	38 (67.9)	18 (32.1)
Revision THA	21 (55.3)	17 (44.7)
Revision TKA	13 (61.9)	8 (38.1)

Correct and incorrect distribution of ChatGPT 4’s performance stratified by question category. *Chi*-square test showed no significant differences between categories (*P* value = 0.255).

The Adult Reconstruction Fellow correctly answered 88.9% of the anatomy questions (8 of 9 questions), 93.3% (28 of 30 questions) in arthroplasty trauma, 83.3% (35 of 42 questions) in basic science, 76.2% (16 of 21 questions) in medical management, 68.8% (11 of 16 questions) in miscellaneous, 78.3% (18 of 23 questions) in PJI, 74.4% (32 of 43 questions) in primary THA, 94.6% (53 of 56 questions) in primary TKA, 84.2% (32 of 38 questions) in revision THA, and 90.5% (19 of 21 questions) in revision TKA, as shown in Table 2. Statistical analysis showed no significant differences in performance across these categories (*P* value = .093).

**Table 2:** Adult Reconstruction Fellow's performance.

Question category	Correct (n = 252) n (%)	Incorrect (n = 47) n (%)
Anatomy	8 (88.9)	1 (11.1)
Arthroplasty trauma	28 (93.3)	2 (6.7)
Basic science	35 (83.3)	7 (16.7)
Medical management	16 (76.2)	5 (23.8)
Miscellaneous	11 (68.8)	5 (31.3)
PJI	18 (78.3)	5 (21.7)
Primary THA	32 (74.4)	11 (25.6)
Primary TKA	53 (94.6)	3 (5.4)
Revision THA	32 (84.2)	6 (15.8)
Revision TKA	19 (90.5)	2 (9.5)

Correct and incorrect distribution of the Adult Reconstruction Fellow's performance stratified by question category. *Chi*-square test showed no significant differences between categories (*P* value = 0.093).

The Adult Reconstruction Attending correctly answered 77.8% of the anatomy questions (7 of 9 questions), 86.7% (26 of 30 questions) in Arthroplasty Trauma, 76.2% (32 of 42 questions) in basic science, 85.7% (18 of 21 questions) in Medical Management, 81.2% (13 of 16 questions) in Miscellaneous, 91.3% (21 of 23 questions) in PJI, 88.4% (38 of 43 questions) in Primary THA, 91.1% (51 of 56 questions) in Primary TKA, 81.6% (31 of 38 questions) in Revision THA, and 85.7% (18 of 21 questions) in Revision TKA, as detailed in Table 3. Statistical analysis revealed no significant differences in performance across these categories (*P* value = .706).

**Table 3:** Adult Reconstruction Attending performance.

Question category	Correct (n = 255) n (%)	Incorrect (n = 44) n (%)
Anatomy	7 (77.8)	2 (22.2)
Arthroplasty trauma	26 (86.7)	4 (13.3)
Basic science	32 (76.2)	10 (23.8)
Medical management	18 (85.7)	3 (14.3)
Miscellaneous	13 (81.2)	3 (18.8)
PJI	21 (91.3)	2 (8.7)
Primary THA	38 (88.4)	5 (11.6)
Primary TKA	51 (91.1)	5 (8.9)
Revision THA	31 (81.6)	7 (18.4)
Revision TKA	18 (85.7)	3 (14.3)

Correct and incorrect distribution of the Adult Reconstruction Attending's performance stratified by question category. *Chi*-square test showed no significant differences between categories (*P* value = 0.706).

### Overall performance of ChatGPT compared to an adult reconstruction fellow and attending

Out of the 299 questions evaluated, ChatGPT 4 correctly answered 200 questions, yielding an accuracy rate of 66.9%. In comparison, the adult reconstruction fellow accurately answered 252 questions (84.3%), and the attending answered 255 questions correctly (85.3%). Statistical analysis revealed no significant differences between the performances of ChatGPT 4 and the fellow (*P* value = .066), as well as between ChatGPT 4 and the attending (*P* value = .399), as detailed in Table 4.

**Table 4:** ChatGPT compared to Adult Reconstruction Orthopaedic Surgeons.

ChatGPT 4			
Adult reconstruction orthopaedic surgeons	Correct n (%)	Incorrect n (%)	Total n (%)
Fellow			
Correct	174 (69.0)	78 (31.0)	252 (84.3)
Incorrect	26 (55.3)	21 (44.7)	47 (15.7)
Attending			
Correct	173 (67.8)	82 (32.3)	255 (85.3)
Incorrect	27 (61.4)	17 (38.6)	44 (14.7)

ChatGPT 4 performance compared to an Adult Reconstruction Fellow and Attending. *Chi*-square test showed no significant differences in performance between ChatGPT 4 and the Adult Reconstruction Fellow and Attending (*P* value = 0.066, and *P* value = 0.399; respectively).

### ChatGPT's accuracy rate on image-based questions

Of the 299 questions assessed, 168 (56.2%) included images, while 131 (43.8%) were text-only. ChatGPT 4 exhibited an accuracy rate of 61.9% (104 of 168 questions) in image-based questions compared to 73.3% (96 of 131 questions) in text-only questions, performing significantly better in text-only questions (*P* value = .038). The Adult Reconstruction Fellow and Attending correctly answered 86.3% (145 of 168 questions), and 85.7% (144 of 168 questions) in questions that contained images, and 81.7% (107 of 131 questions) and 84.7% (111 of 131 questions) in questions that were text-only, respectively. Statistical analysis revealed no significant differences in performance between image and text-only questions for both the Fellow and the Attending (*P* value = .275, *P* value = .812; respectively). These findings are summarized in Table 5.

**Table 5:** Performance Based on the Presence or Absence of Images.

Participant	With image n (%)	Text-only n (%)	*P* Value
ChatGPT 4			.038
Correct	104 (61.9)	96 (73.3)	
Incorrect	64 (38.1)	35 (26.7)	
Fellow			.275
Correct	145 (86.3)	107 (81.7)	
Incorrect	23 (13.7)	24 (18.3)	
Attending			.812
Correct	144 (85.7)	111 (84.7)	
Incorrect	24 (14.3)	20 (15.3)	

Accuracy rate of ChatGPT 4, Adult Reconstruction Fellow, and Attending in questions with or without images. The *Chi*-square test showed a significant difference in ChatGPT 4's accuracy rate in questions with images compared to those without images (*P* value = .038). No significant differences in the Adult Reconstruction Fellow's or Attending's performance in questions with or without images were observed (*P* value = .275, and *P* value = .812; respectively).

When performance on image-based questions was stratified by the type of visual elements, ChatGPT 4 obtained an accuracy rate of 55.0% (11 of 20 questions) in questions with clinical pictures, 33.3 % (1 of 3 questions) with histology, 66.7% (2 of 3 questions) with MRI, 0.0% (0 of 1 questions) with ultrasound, 62.6% (77 of 123 questions) with X-ray, 81.8% (9 of 11 questions) with X-ray and clinical picture, 50.0% (1 of 2 questions) with X-ray and CT, and 60.0% (3 of 5 questions) with X-ray and MRI. The Adult Reconstruction Fellow correctly answered 80.0% (16 of 20 questions) in questions with clinical pictures, 66.7 % (2 of 3 questions) with histology, 66.7% (2 of 3 questions) with MRI, 100.0% (1 of 1 questions) with ultrasound, 87.0% (107 of 123 questions) with X-ray, 90.9% (10 of 11 questions) with X-ray and clinical picture, 100.0% (2 of 2 questions) with X-ray and CT, and 100.0% (5 of 5 questions) with X-ray and MRI. The Adult Reconstruction Attending obtained an accuracy rate of 80.0% (16 of 20 questions) in questions with clinical pictures, 66.7 % (2 of 3 questions) with histology, 100.0% (3 of 3 questions) with MRI, 100.0% (1 of 1 questions) with ultrasound, 86.2% (106 of 123 questions) with X-ray, 90.9% (10 of 11 questions) with X-ray and clinical picture, 100.0% (2 of 2 questions) with X-ray and CT, and 80.0% (4 of 5 questions) with X-ray and MRI. No significant differences in performances based on the type of images were observed, as shown in Table 6 (ChatGPT 4: *P* value = .284; Fellow: *P* value = .769; Attending: *P* value = .946).

**Table 6:** Accuracy Rates based on Types of Images in Questions.

Type of image	ChatGPT 4		Fellow		Attending	
	Correct n (%)	Incorrect n (%)	Correct n (%)	Incorrect n (%)	Correct n (%)	Incorrect n (%)
Clinical Picture	11 (55.0)	9 (45.0)	16 (80.0)	4 (20.0)	16 (80.0)	4 (20.0)
Histology	1 (33.3)	2 (66.7)	2 (66.7)	1 (33.3)	2 (66.7)	1 (33.3)
MRI	2 (66.7)	1 (33.3)	2 (66.7)	1 (33.3)	3 (100.0)	0 (0.0)
Ultrasound	0 (0.0)	1 (100.0)	1 (100.0)	0 (0.0)	1 (100.0)	0 (0.0)
X-ray	77 (62.6)	46 (37.4)	107 (87.0)	16 (13.0)	106 (86.2)	17 (13.8)
X-ray & Clinical Picture	9 (81.8)	2 (18.2)	10 (90.9)	1 (9.1)	10 (90.9)	1 (9.1)
X-ray & CT	1 (50.0)	1 (50.0)	2 (100.0)	0 (0.0)	2 (100.0)	0 (0.0)
X-ray & MRI	3 (60.0)	2 (40.0)	5 (100.0)	0 (0.0)	4 (80.0)	1 (20.0)

Accuracy rate of ChatGPT 4, Adult Reconstruction Fellow and Attending based on the types of images presented in the questions.

*Chi*-square tests showed no significant differences in ChatGPT 4's, Adult Reconstruction Fellow's or Attending's performance between the types of images (*P* value = .284, *P* value = .769, *P* value = .946; respectively).

## Discussion

The integration of AI models like ChatGPT in medical education has demonstrated potential, particularly in orthopaedic surgery. Recently, its application in scientific writing and its capability to address the patient's most common questions have been explored [[4], [5], [6],^8^,[14], [15], [16]]. However, ChatGPT's ability to answer arthroplasty board-style questions and how it compares with an Adult Reconstruction Fellow and a senior-level Fellowship-trained hip and knee arthroplasty attending has not been explored yet. To our knowledge, this is the first study to specifically evaluate ChatGPT's ability to answer arthroplasty questions, including image-based questions. In this study, the performance of ChatGPT 4, Adult Reconstruction Fellow and Attending, were not significantly different when stratified by question category. This is similar to previous findings where ChatGPT 3.5 and 4 performed the same across all subject categories in the ReStudy orthopaedic examination question bank when the subcategories were stratified [8]. Additionally, ChatGPT 4 showed an accuracy rate of 66.9% which was inferior to the performance of the Adult Reconstruction Fellow (84.3%) and Attending (85.3%), but this was not statistically significant. These findings align with the results of *Fiedler et al* where fellowship-trained surgeons outperformed ChatGPT models on the American Shoulder and Elbow Surgeons Maintenance of Certification exam, but the performance gap was not statistically significant in text-only questions [17]. Despite no statistically significant difference in performance, the Adult Reconstruction Fellow and Attending obtained an overall score approximately 18% higher than ChatGPT 4. The observed *P* value of 0.066, while not meeting statistical significance, suggests a meaningful trend in performance differences. These results may warrant further exploration, given the sample size (n = 299), which may have limited the statistical power to detect a true difference. Future studies with larger sample sizes are needed to clarify whether a true difference in performance exists.

In recent studies, the capacity of ChatGPT to interpret medical images, particularly in the context of orthopaedic surgery and arthroplasty, has been critically evaluated. Findings indicate that while ChatGPT 4 can process images, its performance in detailed medical imaging analysis remains suboptimal. Specifically, when answering orthopaedic surgery questions, it exhibited lower performance on image-based questioning, as opposed to text-only inquiries, questioning its current utility in clinical settings [8,18]. Additionally, ChatGPT 4's image interpretation abilities were found to be notably inferior to those of human surgeons, achieving only a 53.2% success rate compared to surgeons' 73.9%, highlighting substantial challenges in the application of multimodal AI to complex medical imaging [17]. Similarly, in our study, ChatGPT 4 performed significantly better in text-only compared to image-based questions and obtained a lower accuracy rate in image-based questions compared to the Adult Reconstruction Fellow and Attending. Unlike AI models, orthopaedic surgeons possess years of clinical experience and practical exposure to radiographs and clinical images, combined with contextual knowledge. The advanced image interpretation skills—such as recognizing visual patterns, integrating clinical context and experience—likely contributed to their higher accuracy in image-based assessments compared to ChatGPT. When our results in the image-based questions were stratified by the different types of images associated with each question, no significant differences were observed in ChatGPT's, or the orthopaedic surgeons’ performances. However, although not statistically significant, ChatGPT obtained the highest accuracy rate in questions that included both, an X-ray and a Clinical Picture. To our knowledge, this is the first study to evaluate ChatGPT's ability to correctly answer image-based questions stratified by the different types of images associated with each question.

Despite some limitations, ChatGPT continues to demonstrate significant utility in medical and patient education. Moreover, ChatGPT may support clinical decision-making by offering academically sourced answers and assisting in preliminary diagnoses of common orthopaedic conditions, though its reliability varies and requires further validation [19,20]. Recent studies have showed that ChatGPT has provided a high accuracy rate in responding to patient's frequent asked questions about THA, TKA, hip arthroscopy, and anterior cruciate ligament surgery [4,16,21,22]. Furthermore, when compared to Google's search outcomes on ulnar collateral ligament injuries, ChatGPT 4 utilized more academic sources and provided more accurate responses, suggesting that it could serve as a more reliable adjunct for clinical information [23]. Another study assessing treatment recommendations for acute hip fractures in elderly patients, ChatGPT 4 showed alignment with the American Academy of Orthopaedic Surgeons Appropriate Use Criteria in multiple cannulated screw fixation, THA, hemiarthroplasty, and long cephalomedullary nails, but it inaccurately assessed treatment appropriateness, underlying limitations in clinical decision-making [24]. Studies consistently point out that while ChatGPT can support medical professionals and enhance patient understanding, it is not yet reliable enough to replace human judgment in clinical settings. These findings contribute to understanding AI's current capabilities and limitations, advocating for its cautious, supplementary use in orthopaedics while underlining the importance of ongoing improvements and validations [9,23].

One of the limitations of this study is that the association of ChatGPT's performance with the requirement of comprehension and application of knowledge to answer questions accurately was not evaluated. Furthermore, the questions were not categorized based on their taxonomic level as described by *Buckwalter et al* [25], which would have provided additional insights into the complexity of the queries addressed. Another limitation is that the assessment of ChatGPT's performance focused solely on the correctness of the answers. Stratification of the questions based on their requirement for simple factual recall vs high-order reasoning was not performed in this study, and is a limitation of our study. Additionally, the logical validity or depth of ChatGPT’s explanations was not examined. As a result, the depth of understanding and the logical coherence of ChatGPT's responses were not assessed, and further studies would be needed to characterize this.

## Conclusions

ChatGPT 4 accurately answered 66.9% of the arthroplasty questions sourced from OrthoBullets, demonstrating a performance below that of an Adult Reconstruction Fellow and Attending, though the differences were not statistically significant. Notably, ChatGPT 4 excelled in text-only questions and showed its best performance in image-based questions when presented with both an X-ray and a clinical picture. These results indicate that while AI can enhance educational tools and provide supportive roles in clinical settings, it is not yet capable of replacing the judgment required for detailed clinical assessments. With ongoing development and validation, ChatGPT could be effectively integrated as a supplementary tool to human expertise in medical practice, ensuring the benefits of AI while upholding the quality of patient care.

## CRediT authorship contribution statement

**Benjamin Nieves-Lopez:** Visualization, Formal analysis, Writing – original draft, Investigation, Project administration, Data curation, Writing – review & editing, Methodology, Conceptualization. **Clayton Wing:** Visualization, Investigation, Writing – original draft, Software, Validation, Data curation, Writing – review & editing, Supervision. **Bryan D. Springer:** Writing – original draft, Software, Data curation, Writing – review & editing, Supervision, Investigation, Visualization, Project administration, Conceptualization, Validation, Methodology. **Keith T. Aziz:** Writing – review & editing, Supervision, Visualization, Resources, Formal analysis, Writing – original draft, Software, Investigation, Validation, Project administration, Data curation, Methodology, Conceptualization.

## Conflicts of interest

Bryan D.Springer receives royalties from Stryker and OsteoRemedies; is a paid consultant for Convatec; owns stock or stock options in Osteal; and has board member/committee appointments at The Hip Society and IOEN.

Keith T. Aziz has board member/committee appointments at the American Society for Surgery of the Hand the Ethics and Professionalism Committee.

The other author declares there are no conflicts of interest.

For full disclosure statements refer to https://doi.org/10.1016/j.artd.2025.101772.

## Appendix A Supplementary data

Conflict of Interest Statement for Springer

Conflict of Interest Statement for Nieves-Lopez

Conflict of Interest Statement for Wing

Conflict of Interest Statement for Aziz
